# Differentiation Capacity of Human Urine-Derived Stem Cells to Retain Telomerase Activity

**DOI:** 10.3389/fcell.2022.890574

**Published:** 2022-05-23

**Authors:** Yingai Shi, Guihua Liu, Rongpei Wu, David L. Mack, Xiuzhi Susan Sun, Joshua Maxwell, Xuan Guan, Anthony Atala, Yuanyuan Zhang

**Affiliations:** ^1^ Institute for Regenerative Medicine, Wake Forest University, Winston-Salem, NC, United States; ^2^ Key Laboratory of Pathobiology, Ministry of Education, College of Basic Medical Sciences, Jilin University, Jilin, China; ^3^ Reproductive Medical Center, Sixth Affiliated Hospital, Sun Yat-Sen University, Guangzhou, China; ^4^ Department of Urology, The First Affiliated Hospital, Sun Yat-Sen University, Guangzhou, China; ^5^ Department of Rehabilitation Medicine and Institute for Stem Cell and Regenerative Medicine, University of Washington, Seattle, WA, United States; ^6^ Bio-Materials and Technology Lab, Grain Science and Industry, Bio and Agricultural Engineering, Kansas State University, Kansas, KS, United States; ^7^ Cardiovascular Disease AdvenHealth Orland, Orland, FL, United States

**Keywords:** telomerase, urine-derived stem cells, longevity, tissue regeneration, differentiation

## Abstract

Telomerase activity is essential for the self-renewal and potential of embryonic, induced pluripotent, and cancer stem cells, as well as a few somatic stem cells, such as human urine-derived stem cells (USCs). However, it remains unclear how telomerase activity affects the regeneration potential of somatic stem cells. The objective of this study was to determine the regenerative significance of telomerase activity, particularly to retain cell surface marker expression, multipotent differentiation capability, chromosomal stability, and *in vivo* tumorigenic transformation, in each clonal population of human primary USCs. In total, 117 USC specimens from 10 healthy male adults (25–57 years of age) were obtained. Polymerase chain reaction amplification of a telomeric repeat was used to detect USCs with positive telomerase activity (USCs^TA+^). A total of 80 USCs^TA+^ (70.2%) were identified from 117 USC clones, but they were not detected in the paired normal bladder smooth muscle cell and bone marrow stromal cell specimens. In the 20–40 years age group, approximately 75% of USC clones displayed positive telomerase activity, whereas in the 50 years age group, 59.2% of the USC clones expressed positive telomerase activity. USCs^TA+^ extended to passage 16, underwent 62.0 ± 4.8 population doublings, produced more cells, and were superior for osteogenic, myogenic, and uroepithelial differentiation compared to USCs^TA−^. Importantly, USCs displayed normal chromosome and no oncological transformation after being implanted *in vivo*. Overall, as a safe cell source, telomerase-positive USCs have a robust regenerative potential in cell proliferation and multipotent differentiation capacity.

## 1 Introduction

Telomerase activity (TA) is closely related to the longevity of pluripotent stem cells (Huang et al., 2014; Li et al., 2020), embryonic stem cells (ESCs) (Hiyama and Hiyama, 2007), induced pluripotent stem cells (iPSCs) (Wang et al., 2012), and tumor cells (Hiyama and Hiyama, 2007). In normal somatic cells, the activity of telomerase extends telomeric repeats and is usually reduced after birth. Interestingly, TA often cannot be detected in most human mesenchymal stem cells (MSCs) (Zimmermann et al., 2003; Hiyama and Hiyama, 2007), whereas low levels of telomerase are identified in some somatic stem cells from the hematopoietic system (Thongon et al., 2021), intestinal mucosa, and epidermal basal layers (Hiyama and Hiyama, 2007). Human MSCs, such as bone marrow-derived stem cells (BMSCs) (Bernardo et al., 2007; Hiyama and Hiyama, 2007), adipose-derived stem cells (ASCs) (Nava et al., 2015b), and skeletal muscle progenitor cells (SMPCs), often display telomerase negativity, although these stem cells have the MSC phenotype (SH2^+^, SH3^+^, SH4^+^, CD29^+^, CD44^+^, CD14^−^, CD34^−^, and CD45^−^) and can differentiate into adipocytes, chondrocytes, and osteoblasts (Zimmermann et al., 2003). A possible reason for the negative telomerase result is the occurrence of alternative lengthening of telomeres (ALT) in MSCs, which is an alternative telomere length-maintaining mechanism (Lafferty-Whyte et al., 2009).

Our previous study was the first to demonstrate that progenitor/stem cells exist in the urine, thus we proposed the name urine-derived stem cells (USCs) (Zhang et al., 2008; Bodin et al., 2010; Wu et al., 2011; Bharadwaj et al., 2013). These cells can be easily isolated from urine samples via a non-invasive approach (Kang et al., 2015), which offers clear advantages over the stem cells harvested from other sources, like bone marrow or adipose aspirates. Clonal USC populations can be readily generated from a single cell by limiting dilution of the starting mixed culture. Each micro-colony will proliferate into a clonal population with many cells (approximately 6.4 × 10^7^ cells) within 4 weeks. Urine collected over 24 h from one individual will generate approximately 140 clones (or 10 clones/200 mL urine), which will expand to greater than 1 × 10^8^ cells by passage three within 3 weeks. Cells do not require tissue dissociation procedures with digestive enzymes, which help to preserve cell viability (Lang et al., 2013). Despite their proliferative capacity, USCs display low expression of gene markers of stem cells (SOX2, OCT3/4, c-MYC, and KLF4) (Bharadwaj et al., 2013) whereas providing a robust proliferative potential reaching up to p16 in a >60-day culture. Understanding the impact of TA on cellular proliferation *in vitro* and the potential regenerative capacity *in vivo* is essential to evaluate the suitability of using USCs for tissue repair, disease modeling, and drug development.

It is well known that telomerase activity is critical for the self-renewal or cell proliferation capacity in stem cells, however, the role of TA in multiple differentiation potential is controversial. The aim of this study was to determine the role of TA in maintaining stemness with cell longevity, proliferation capacity, multipotent differentiation potential, cell surface marker expression, karyotype stability, and the risk of *in vivo* teratoma formation in human primary urine-derived stem cells (USCs).

## 2 Materials and Methods

### 2.1 Collection and Culture of Urine-Derived Stem Cells

This study was approval by the Wake Forest University Health Sciences Institutional Review Board. A total of 50 urine samples were collected from 10 healthy male individuals ranging from 25 to 57 years of age and divided into four age groups (20, 30, 40, 50 years of age). A total of 117 USCs clones were isolated, expanded, and characterized as previously described (Zhang et al., 2008; Bodin et al., 2010; Wu et al., 2011). Briefly, USCs were grown in culture media composed of keratinocyte serum-free medium (KSFM) and embryonic fibroblast medium (EFM) mixed at a ratio of 1:1 (Zhang et al., 2008). Only wells in 24 plates that contained single cell were scored and used for further experimentation.

### 2.2 Telomerase Activity Assay

Telomerase activity levels were measured with the Telo *TAGGG* Telomerase PCR ELISA plus kit (Roche Applied Science, Mannheim, Germany), according to the manufacturer’s recommendations. HEK 293 cells were used as a positive control, and human BMSC and smooth muscle cells (SMC) were used as negative controls. Briefly, 2 × 10^5^ cells at passage two were collected after being trypsinized and washed with cold PBS. Telomerase added the telomeric repeats (TTAGGG) in the kit to the 3’ end of the biotin-labeled synthetic P1-TS primer. These elongated products, and the internal standard (IS) included in the same reaction vessel, were amplified by PCR using the primers P1-TS and the anchor-primer P2. The PCR products were divided into two aliquots, denatured, and hybridized separately to digoxigenin (DIG) labeled detection probes specific for the telomeric repeats and for IS (P3-Std). The resulting products were immobilized via the biotin label onto a streptavidin-coated microplate. Immobilized amplicons were detected with an antibody against digoxigenin that is conjugated to horseradish peroxidase (Anti-DIG-HRP) and the sensitive peroxidase substrate TMB. Absorbance values were measured as the A_450nm_ reading against a blank (reference wavelength A_690nm_) by using a spectrophotometer. Relative telomerase activity (RTA) within different samples in an experiment were obtained using the following formula (Kim et al., 1994; Kim and Wu, 1997):RTA = [(AS–AS, O)/AS, IS]/[(ATS, 8–ATS8, 0)/ATS8, IS]] × 100.

AS: absorbance of sample; AS, 0: absorbance of heat-treated sample; AS, IS: absorbance of Internal standard (IS) of the sample; ATS8: absorbance of control template (TS8); ATS8, 0: absorbance of lysis buffer; ATS8, IS: absorbance of Internal standard (IS) of the control template (TS8). The kit included the IS and TS8.

To further determine the influence of time on telomerase activity, the RTA of two pairs of USCs (age group 20–50 years), telomerase activity positive (TA^+^) and telomerase activity negative (TA^−^), after every five passages or the end passage were measured by the above protocol (Kim et al., 1994).

### 2.3 Cell Proliferation

The USCs in passage three were seeded in 96-well plates at a density of 2,500 cells/well. The culture medium was replaced every second day. Cell proliferation was determined on days 1, 3, 5, and 7 using an MTS cell proliferation assay kit (CellTiter 96^®^ AQueous One Solution Cell Proliferation Assay, Promega) according to the manufacturer’s instructions. Briefly, the MTS reagent was incubated with the cells in the dark for 1 h at 37°C. Following incubation, the absorbance was measured at 490 nm using a spectrophotometer (Molecular Devices Inc., Sunnyvale, CA, United States ). Triplicate measurements were conducted for each time point. The population doubling (PD) and doubling time (DT) calculations were determined based on single USCs at p0 up to the maximum passage (p16) for each clone. The USCs were trypsinized when they reached 70–80% confluence, and the cells were counted manually using a hemocytometer. The PD and DT were calculated using the following formula (Bharadwaj et al., 2011; Bharadwaj et al., 2013):

PD = ln (N_f_/N_i_)/ln (2) and DT = C_t_/PD

N_f_ is the final number of cells, N_i_ is the initial number of cells, and C_t_ is the culture time.

### 2.4 Flow Cytometry

Flow cytometry analysis for the USCs involved staining the USCs with specific labeled anti-human antibodies: CD25-PE, CD31-FITC, CD34-FITC, CD44-FITC, CD45-FITC, CD73-PE, CD90-FITC, CD105-PerCP-CY5.5, CD117-PE, CD140b-PE, and CD146-PE (BD Pharmingen™). Briefly, USCs (p4) were trypsinized, and 5.0 × 10^5^ cells were washed with pre-chilled PBS containing 1% bovine serum albumin (BSA). The fluorescence conjugated antibodies listed above were incubated with USCs on ice for 30 min in the dark. IgG1-PE, IgG1-FITC, IgG2b-FITC, and IgG1-PerCP-Cy^TM^5.5-conjugated isotype control antibodies were used to determine background fluorescence. Cells were washed twice with wash buffer, passed through a 70 µm filter, and analyzed using FACSCalibur™ flow cytometry (BD Biosciences, Franklin Lakes, NJ, United States).

### 2.5 Multipotent Differentiation of USCs *In Vitro*

To determine the differentiation capacity difference between USCs^TA+^ and USCs^TA−^, cells were subjected to the following induction described below, and their changes in morphology and/or histochemical staining for specific components were recorded.

Smooth muscle cell induction: Three pairs of USCs^TA+^ and USCs^TA−^ (p3) from different age groups were plated at 2,000 cells/cm^2^ in smooth muscle differentiation media containing equal amounts of DMEM (high glucose) and EFM with 10% fetal bovine serum (FBS) and 2.5 ng/mL TGF-β1 and 5 ng/mL PDGF-BB (R&D Systems, Minneapolis, MN, United States). Cell morphology was recorded before and after growth factor additions for up to 14 days.

Uroepithelial induction: Three pairs of USCs^TA+^ and USCs ^TA−^ (p3) were plated at 3,000 cells/cm^2^ in a medium containing equal amounts of KSFM and EFM with 2% FBS and 30 ng/mL EGF (R&D Systems, Minneapolis, MN, United States ) mixed with the conditional medium of urothelial cells (UC, 1:1) for 14 days.

Osteogenic induction: USCs (p3) were seeded at a density of 4,000 cells/cm^2^ and cultured in serum containing DMEM low-glucose medium with 100 nM dexamethasone, 10 mM β-glycerophosphate and 50 mM ascorbic acid-2-phosphate (Wako Chemicals, Richmond, VA, United States ) for 28 days. The induced cells were harvested after 28 days and fixed in 95% ethanol before histochemical staining. For detection of calcium secreted by the osteogenic-differentiated cells, Alizarin Red S staining was conducted. Briefly, the fixed cells were incubated with 0.5% Alizarin Red S dye (pH 4.1) to sufficiently cover the cell layer for 3–5 mins. Excess dye was removed with distilled water before photo documentation.

### 2.6 Western Blotting

Proteins of SMC and UC-induced USCs^TA+^ and USCs ^TA−^ and were extracted using RIPA lysis buffer with a proteinase inhibitor cocktail (Complete mini; Roche Applied Sciences). Protein samples (15–30 µg) were analyzed on a 6–12% SDS-PAGE, and after electrophoresis the proteins were transferred to PVDF membranes (Millipore, Billerica, MA, United States ). The membranes were blocked with skimmed milk and incubated with the primary antibody at an appropriate dilution overnight at 4°C. After secondary antibody incubation, hybridization was detected using the Western Lightning Chemiluminescence reagent (PE, Waltham, MA, United States).

### 2.7 Permeability Assay

To assess the barrier function of urothelial-differentiated USCs^TA+^ and USCs^TA−^, non-induced USCs^TA+^ and USCs^TA−^, SMCs, and ureter UCs, these cells were cultured on 0.4 µm Transwell inserts (353,090, Becton Dickinson) placed in 6-well dishes as previously reported (Cross et al., 2005) with minor modifications. Briefly, the inserts were coated with collagen IV (3 µg/cm^2^) and air-dried in a laminar hood. Cells (1 × 10^5^/cm^2^) were plated in 1.5 mL of 1 mg/mL tracer-containing medium (FITC-dextran, 4 kDa, Sigma, FD4) in the insert (top chamber) and 3 mL tracer-free medium in the bottom well. Phenol-free medium was used to avoid interference from the indicator in the assay. A day before the tracer was added, the media were supplemented with 2 mM CaCl_2_ solution. After 3 h, 100 µL media aliquots were removed for fluorescence measurements (excitation at 490 nm and emission at 520 nm). Results were plotted as a percentage relative to the “no cell” control.

### 2.8 Karyotype Analysis

To determine the chromosomal stability of cultured USCs, the karyotypes of early (p4) and late passage (TA^+^ clone at p12, TA^−^ clone at p8) cells were measured (Ge et al., 2011). Cultured cells were trypsinized with a 0.25% Trypsin-EDTA solution, resuspended in hypotonic solution (0.075 M KCl) and then fixed with methanol/acetic acid solution in a 3:1 proportion. The metaphase spread on glass slides was digested by trypsin and then stained with Giemsa stain to generate G bands along each chromosome. Standard cytogenetic analysis was performed under microscopy. Chromosomal image capture and karyotyping were performed using CytoVision^®^, version 3.7.

### 2.9 Soft Agar Assay in Vitro

Agar assays are often used to distinguish tumor cells from non-transformed or normal cells because normal cells cannot undergo anchorage-independent growth or thrive on an agar substrate. To evaluate whether both USCs^TA+^ and USCs^TA−^ induce tumorigenicity, USCs were tested on agar gels. HeLa cells and SMC were used as positive and negative controls, respectively. Briefly, 0.35% upper agar layer and 4% base agar layer were prepared in 35 mm tissue-treated dishes. Cells were seeded at the top of the upper agar at a density of 5,000 cells/well. Culture medium was changed twice a week. Cell morphology, proliferation rate, anchorage-independent growth, and cell colony formation were observed under a phase contrast microscope. After culturing for 2 weeks, all the samples were stained with 1 mL of 0.05% nitroblue tetrazolium (NBT) prepared in PBS and sterilized (0.2 micron). This was incubated overnight at 37°C. The cells that took up NBT and showed a violet color were determined to be live cells.

### 2.10 Spontaneous Transformation *In Vivo* Assay

Experiments using nude mice were approved by the Wake Forest University Health Sciences Institutional Animal Care and Use Committee. To further determine the non-tumorigenicity of USCs^TA+^ *in vivo*, two USCs^TA+^ (p5) were implanted in the kidney subcapsular region of NSG (NOD.Cg-Prkdcscid-Il2rgtm1Wjl/SzJ) mice. H9 (human embryonic stem cell line) was used as a positive control. A total of 12 six-week-old female NSG mice (Jackson Labs, Bar Harbor) were used and divided into two groups: USC^TA+^ (3 mice/clone) and H9 groups (6 mice/group). In total, 2 × 10^6^ cells in 20 µl of PBS were injected into the subcapsular region of the right kidney. After 8 weeks, the mice were sacrificed and bilateral kidneys were fixed in 4% paraformaldehyde, dehydrated, and embedded in paraffin. For evaluation of general graft histology and teratoma formation, routine hematoxylin and eosin (H&E) staining was performed.

## 3 Results

### 3.1 Telomerase Activity Detected in USCs at Early Passage

**FIGURE 1:**
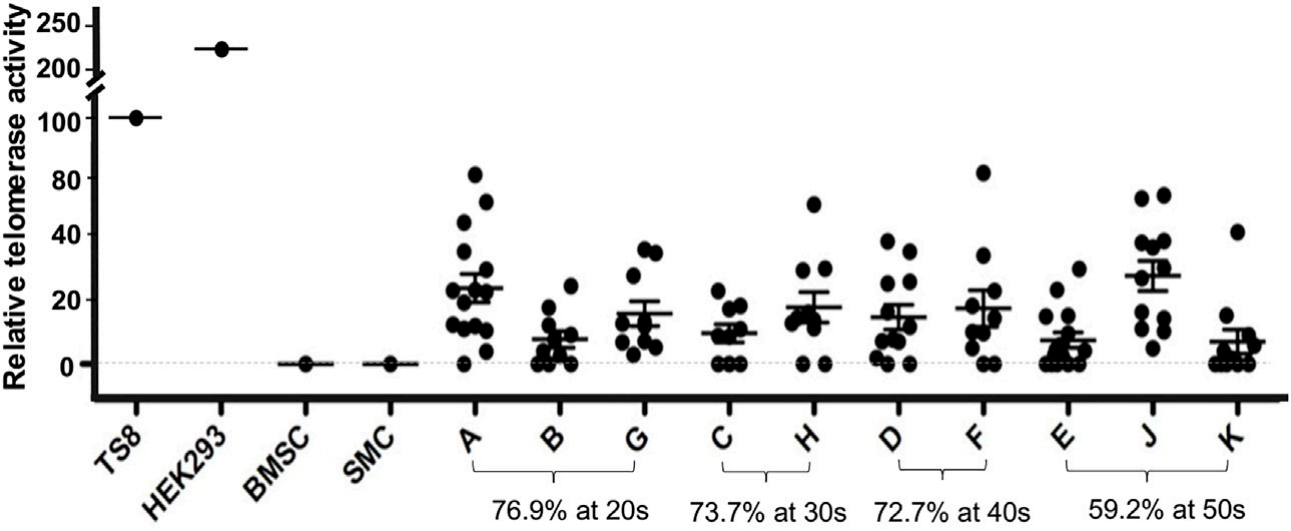
Distribution of human USCs^TA+^ in different age groups. Most USCs (76.9–72.7%) retained telomerase activity in donors at age 20–40 years of age, but the ratio of USCs^TA+^ significantly dropped to 59.2% in the donors over 50 years. Cell extracts were prepared from 2 × 10^5^ cells and assayed for telomerase activity, according to the manufacturer’s instructions (Telo *TAAGG* ELISAPLUS kit Roche). TS8 was provided as a representative positive control lysate prepared from HEK-293 cells. BMSC; human mesenchymal stromal cells of bone marrow, SMC; smooth muscle cells as controls, and A, B, C, D, E, F, G, H, J, K; individual USC clones from different aged healthy individuals (20–50 s), at passage 2 (p2). RTA is the percentage (%) of TS8 (internal positive control) after being normalized.

A total of 80/117 USC clones (p2) from 10 individuals showed detectable TA (70.2% were USCs^TA+^). There were no significant differences in the positive rate for TA in the 20–40 years age group: 20 s: 30/39 (76.9%), 30s: 14/19 (73.7%), and 40s: 16/22 (72.7%) represented USC clones, respectively, but the RTA notably decreased to 16/27 (59.2%) in the 50 years age group, as shown in Figure 1 and Table 1. TA could not be detected in human BMSCs at p2.

**TABLE 1:** The rate, mean, and highest telomerase activity of the isolated USCs^TA+^ clones for individual donors (p2) in the four different age groups.

Donor’s ages (no. of donors)	Donors (no. of clones)	No. of USCTA + clones (% in total USC	Mean TA ± SD (%)	Highest RTA
20s (*n* = 3)	A (*n* = 19)	16 (84.2%)	24.38 ± 14.41	53.25
	B (*n* = 10)	6 (60.0%)	14.94 ± 7.93	25.94
	G (*n* = 10)	8 (80.0%) 76.9%	16.85 ± 10.86	32.24
30s (*n* = 2)	C (*n* = 9)	6 (66.6%)	12.98 ± 5.28	20.56
	H (*n* = 10)	8 (80.0%) 73.7%	19.94 ± 12.05	44.93
40s (*n* = 2)	D (*n* = 12)	9 (75.0%)	17.41 ± 10.99	34.50
	F (*n* = 10)	7 (70.0%) 72.7%	21.69 ± 16.03	53.75
50s (*n* = 3)	E (*n* = 17)	7 (41.2%)	18.55 ± 12.37	41.36
	J (*n* = 10)	9 (90.0%)	26.67 ± 13.84	47.49
	K (*n* = 10)	4 (40.0%) 59.2%	16.03 ± 14.50	37.12

Notes: Telomerase activity was expressed as a percentage of the RTA, of USCs, to the RTA, of TS8. The suffixes A, B, G, C, H, D, E, F, J, K represent the ten healthy individuals who participated in the study.

**FIGURE 2:**
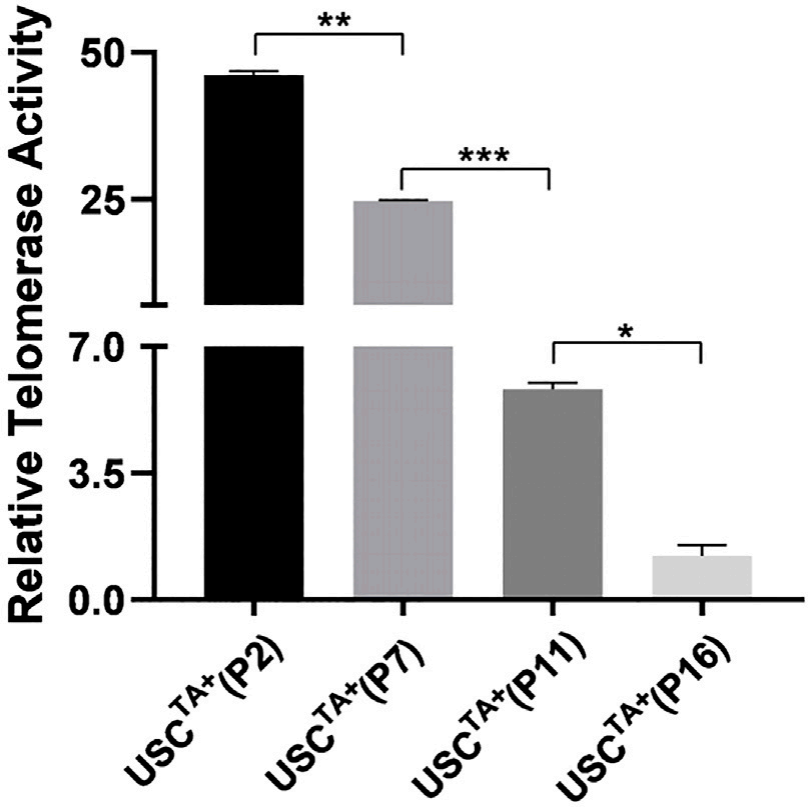
Strength of telomerase activity declined over passage of cultured USCs. Telomerase activity of USC^TA+^ clones (A35 and J29) from donors of 20 and 50 years of age were determined in different passages (p2, p7, p11, and the last passage). The relative telomerase activity (RTA) of USC^TA+^ clones declined gradually in the passages during culture *in vitro*.

To determine if the TA in human USCs decreased as the passage progressed, we measured the TA of two pairs of USCs^TA+^ and USCs^TA−^ at early passage (p2) and late passages (p7, p11 or the last passage for USCs^TA+^) from the 20s and 50s groups. The strength of RTA of USC^TA+^ clones decreased gradually with passage (Figure 2), which seems to make a formerly TA+ clone equivalent to a TA^−^clone. The RTA remained at undetectable levels through all the passages of USC^TA−^ clones.

### 3.2 Self-Renewal Ability of USCs^TA+^ Generated More Cells

**FIGURE 3:**
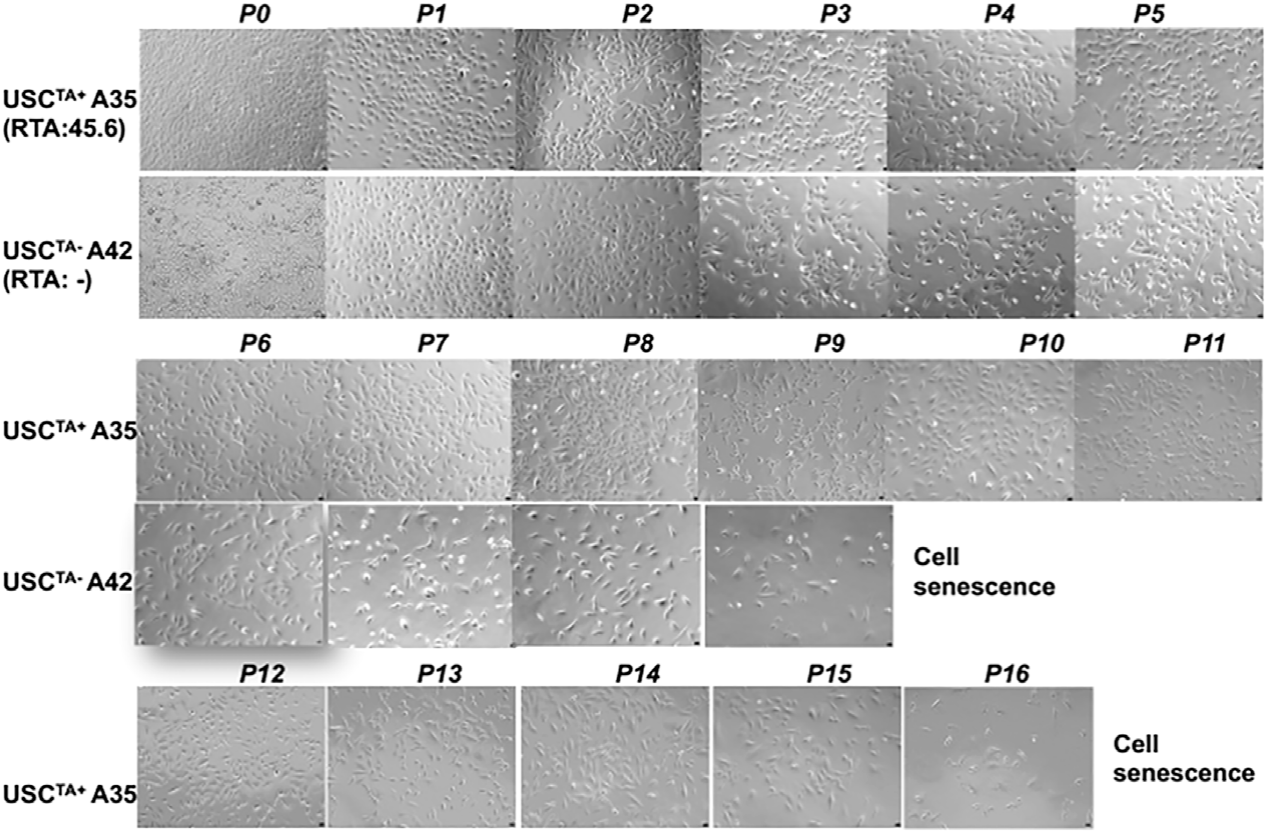
Changes in the morphologic appearance of USCs^TA+^ and USCs^TA−^ at different passages. A USC^TA+^ clone (A35, RTA: 45.6, from the donor at 20 years of age) maintained the “rice-grain”-like appearance up to p16; a USC^TA-^ clone (A42, RTA: negative, from the same donor at an age of 20 years of age) showed a flattened cell shape or cells of larger size, and stopped growing (cell senescence) at p9, assessed by phase contrast microscopy. Magnification, ×100.

The USC^TA+^ and USCs^TA−^ paired clones from a single donor were tested in parallel to prevent other factors from affecting the results of the comparison. The USC^TA+^ clone A35 which was isolated from a 27-year-old male donor, could be passed more than the USCs^TA−^ clone A42 which was isolated from the same donor, and consistently maintained the original “rice-grain like” morphology until they reached cell senescence at p16. Similarly, the USC^TA−^ clones steadily displayed the similar cell morphologic appearances and finally displayed a larger, flattened, and the typical “fried egg” morphology of quiescent cells at p9 (Figure 3).

**FIGURE 4:**
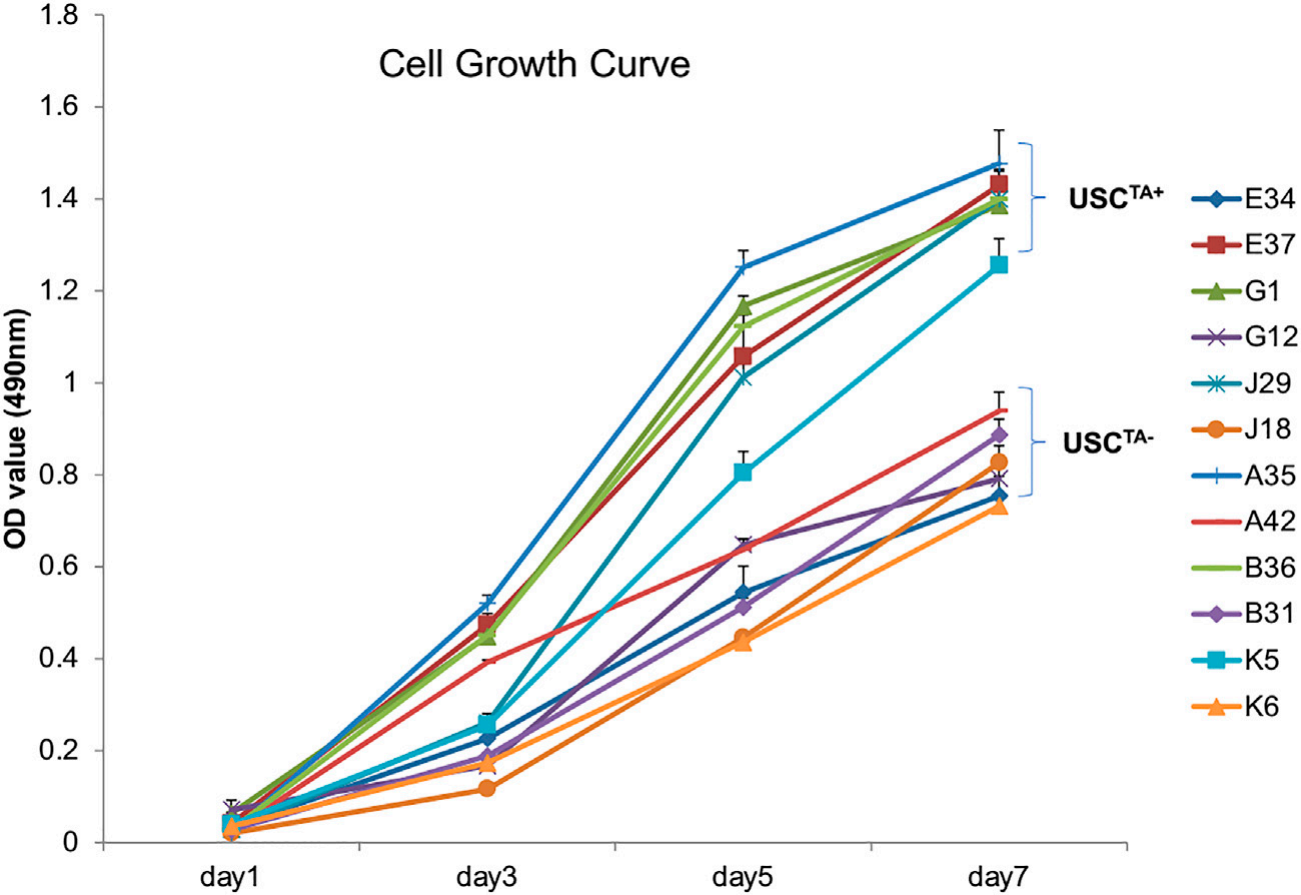
Cell growth curve of USCs^TA+^ vs. USCs^TA−^. Cell proliferation was measured as the USCs were cultured for 7 days. Six individual USC^TA+^ clones (*n* = 6, p3, A35, B36, G1, E37, J29, and K5) generated significantly more cells and grew faster than USC^TA−^ clones (*n* = 6, p3, A42, B31, G12, E34, J18, and K6).

**FIGURE 5:**
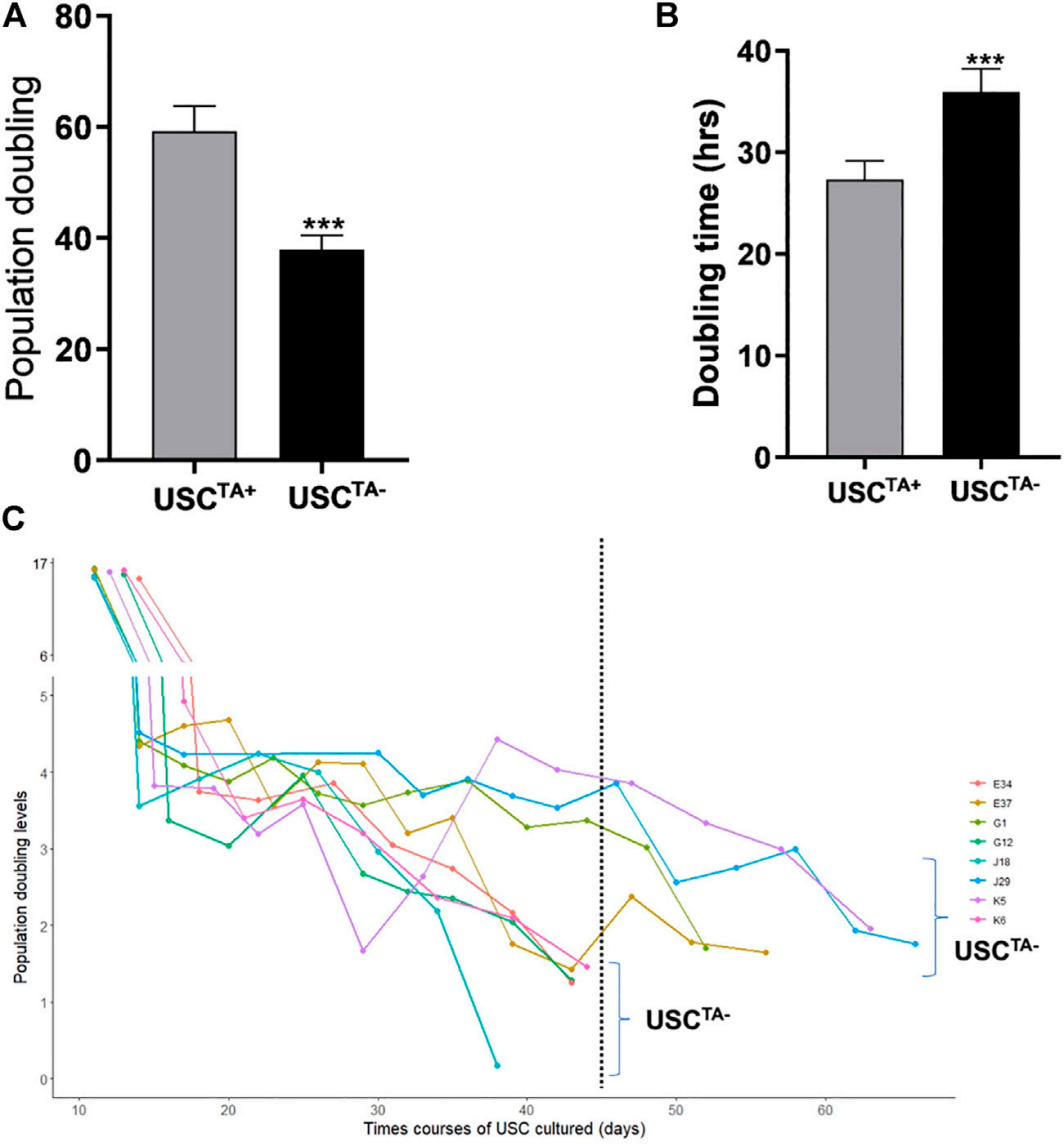
Population doubling, doubling time, and *in vitro* survival time course of USCs^TA+^ vs USCs^TA−^. **(A)** Population doubling significantly increased for USCs^TA+^ compared to USCs^TA−^. **(B)** Doubling time was significantly shorter in USCs^TA+^ than in USCs^TA−^. **(C)** Cellular senescence in USCs^TA−^ occurred earlier than that in USCs^TA+^ upon cell expansion. These data indicate that USCs^TA+^ proliferates more rapidly, generates more cells, and survives longer than USCs^TA−^. USCs from healthy individuals (*n* = 12) were cultured following plating at a single cell/well. Six individual USC^TA+^ clones (*n* = 6, p3, A35, B36, G1, E37, J29, and K5) generated significantly more cells and grew faster than USC^TA−^ clones (*n* = 6, p3, A42, B31, G12, E34, J18, and K6). USC^TA+^ clones survived longer and had longer population doubling times. The suffixes A, B, G, C, H, D, E, F, J, and K represent the ten healthy individuals who participated in the study.

According to the cell growth curve data (Figure 4), the USC^TA+^ clones grew more rapidly than the USC^TA−^ clones. Consequently, the PD of USC^TA+^ clones increased significantly compared to that of the USC^TA−^ clones, regardless of the individual’s age (*p <* 0.001). The mean DTs of USC^TA+^ clones were significantly shorter than those of the USC^TA−^ clones (Table 2; Figure 5) (*p <* 0.001).

**TABLE 2:** Population doubling and doubling time of USC^TA+^ vs USC^TA−^ in the 20 and 50 years age groups in early and late passages to determine if there was a difference in telomerase activity with increasing age.

Individual clones (s)			Age (yrs)	RTA (%)	Population Doubling		Doubling Time	
					PD	M ± SD	DT (hrs.)	M ± SD
20s	USC^TA+^ (n = 3)	A35	27	45.6	67.5	62.0 ± 4.8^*^	27.1	26.1 ± 1.6^**^
		G1	25	25.9	59.2	—	24.3	—
		B36	28	32.2	59.2	—	27.0	—
	USC^TA−^(n = 3)	A42	27	—	42.0	39.6 ± 2.6	37.3	35.2 ± 2.4
		G12	25	—	36.8	—	35.7	—
		B31	28	—	40.1	—	32.7	—
50s	USC^TA+^ (n = 3)	E37	50	41.4	55.5	56.6 ± 2.2^*^	29.2	28.5 ± 1.3^**^
		J29	55	46.6	59.2	—	27.0	—
		K5	57	37.1	55.2	—	29.3	—
	USC^TA−^(n = 3)	E34	50	—	35.6	36.3 ± 0.9	36.6	36.6 ± 2.5
		J18	55	—	36.2	—	34.2	—
		K6	57	—	37.2	—	39.1	—

^*^ *p* < 0.001: USC^TA+^ vs USC^TA−^ in, population doubling at age of 20s, and 50s groups, respectively.

^**^ *p < 0.01*: USC^TA+^ vs USC^TA−^ in, doubling time at age of 20s, and 50s groups, respectively. The suffixes A, B, G, C, H, D, E, F, J, and K represent the ten healthy individuals who participated in the study.

However, the relative TA of USCs was not completely coordinated with the cell proliferation capacity (PD, DT, and passage) (Table 2).

### 3.3 Both USC^TA+^ and USC^TA−^ Clones Showed Similar Cell Surface Markers

**FIGURE 6:**
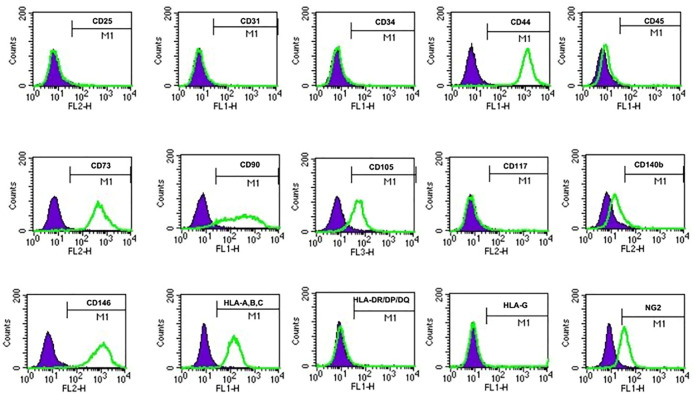
USCs expressing mesenchymal stem cell surface markers. USC^TA+^ clones (A35, B36, G1, E37, J29, and K5) and USC^TA−^ clones (A42, B31, G12, E34, J18, and K6) both displayed sets of mesenchymal stem cell (MSC) surface markers (CD44, CD90, CD73, CD105, and CD146), positive at similar levels, whereas both USCs^TA+^ and USCs^TA−^ did not express sets of haemopoietic stem cell markers (i.e., CD31, CD34, CD45, CD25, and CD117). However, three of five USCs^TA+^ displayed CD105 expression, but no or weak expression in all the four USC^TA−^ cell clones. MSC surface markers of USCs at p4 were detected *via* flow cytometry.

To identify the cell surface markers of USC clones, six pairs of USCs^TA+^ and USCs^TA−^ were subjected to flow cytometry analysis. All the USC clones showed strong positive MSCs markers including CD44, CD90, CD73, CD105, and CD146 and were negative for hematopoietic stem cell markers including CD25, CD31, CD34, CD45, and CD117 (Figure 6; Table 3). However, there was no significant difference in the CD105 expression between USCs^TA+^ and USCs^TA−^.

**TABLE 3:** Cell surface markers of USC^TA+^ and USC^TA−^ clones at passage four detected using fluorescence-activated cell sorting.

CLONES (s)			Cell surface markers (%)														
			CD 25	CD 31	CD 34	CD 44	CD 45	CD 73	CD 90	**CD 105**	CD 117	CD 140b	CD 146	NG2	HLA-a,b,c	HLA-DR/DP/DQ	HLA-G
**20s**	TA+	A35	0.36	0.51	0.40	99.9	0.34	99.8	93.4	**82.2**	0.5	3.9	99.89	3.05	99.97	0.81	4.53
	TA−	A42	0.69	0.81	0.98	100	0.54	99.9	95.1	**5.3**	0.6	7.7	99.86	29.34	99.98	1.18	0.30
	TA+	G1	0.37	0.09	0.07	100	0.09	99.86	90.5	**90.7**	0.4	5.0	99.96	88.56	—	—	—
	TA−	G12	0.04	0.39	0.20	99.7	0.02	97.70	99.8	**99.0**	0.9	0.1	99.67	0.44	—	—	—
	TA+	B36	0.21	0.52	0.60	96.8	0.77	99.73	99.6	**33.0**	0.8	12.0	99.08	15.48	99.69	0.67	0.86
	TA−	B31	0.60	0.61	0.55	99.8	0.93	99.91	99.8	**1.7**	0.5	9.3	99.66	29.56	99.90	0.64	0.84
**50s**	TA+	E37	1.37	0.86	0.52	99.8	0.98	99.97	98.9	**66.1**	0.93	0.1	99.26	5.00	—	—	—
	TA−	E34	3.45	0.77	0.99	100	0.70	99.98	99.7	**91.4**	1.41	0.2	99.76	25.20	—	—	—
	TA+	J29	0.41	0.33	0.16	99.94	0.37	100	92.12	**55.44**	0.31	5.98	98.12	3.06	99.97	0.13	1.09
	TA−	J18	0.45	0.55	0.53	98.36	0.41	95.61	95.61	**3.50**	0.44	0.62	96.51	59.35	98.40	1.06	1.73
	TA+	K5	0.46	0.33	0.26	99.85	0.35	99.90	83.20	**48.67**	0.41	8.71	99.14	64.55	99.70	0.48	1.90
	TA−	K6	0.71	0.47	0.26	99.57	0.32	89.84	49.13	**15.25**	0.70	8.39	99.41	71.18	99.20	0.37	1.42

### 3.4 Potent Differentiation Capacity of USCs^TA+^

**FIGURE 7:**
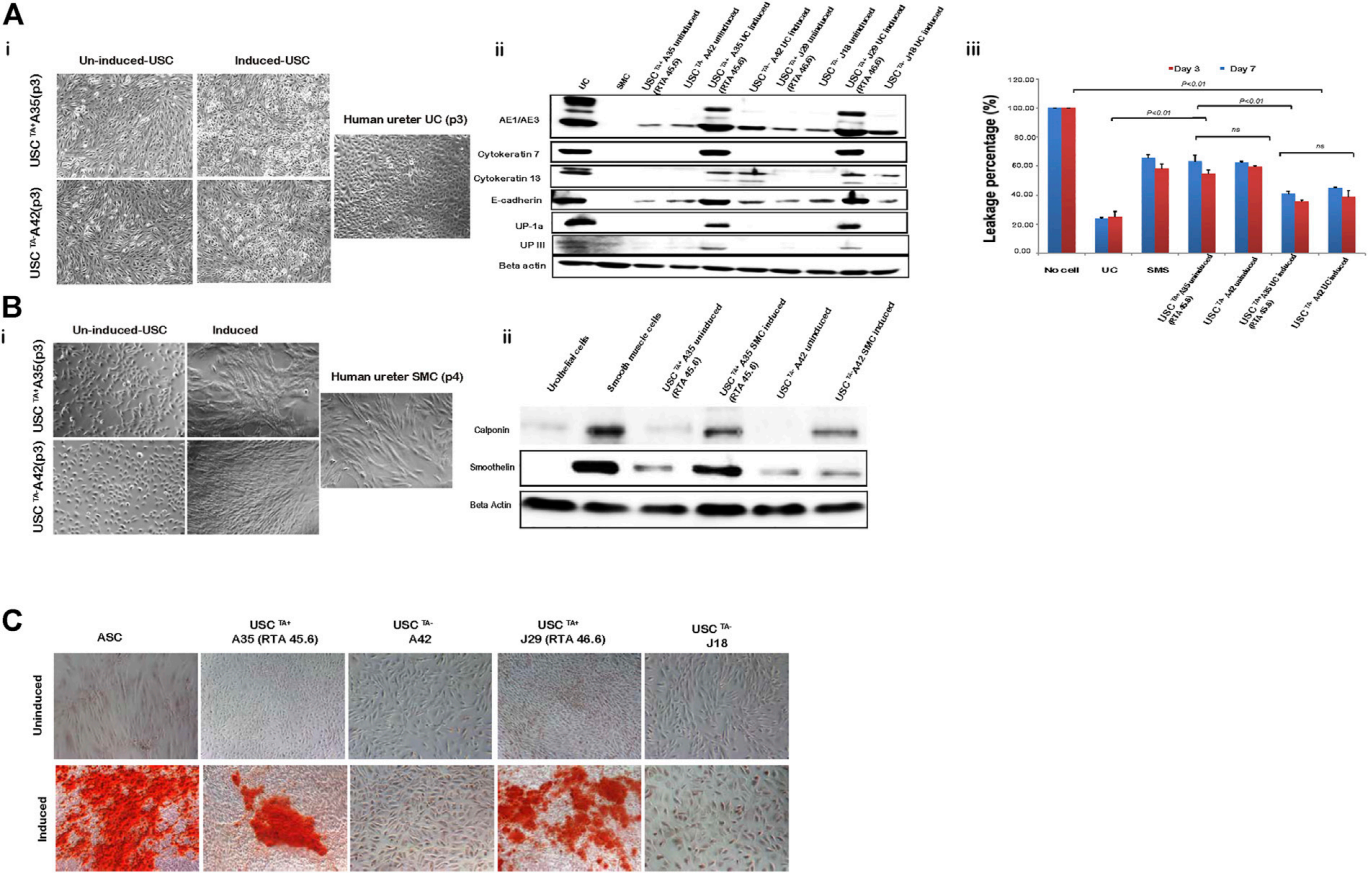
USCs undergo multi-potential differentiation *in vitro*. **(A)**. Morphology of USCs after induction with USC induction media for 14 days. Cell morphology changed from “rice-grain-like” to a cuboidal morphology appearance. Human ureter urothelial cells (UC) were included as a positive control. Urothelial-specific proteins (Uroplakin-Ia, -III, AE1/AE3, E-cadherin, Cytokeratin 7, and Cytokeratin 13) were detected via western blotting. Specific signals (bands) were observed in lanes with proteins from induced USCs^TA+^ and UCs. Barrier function analysis was performed on both USCs^TA+^ and USCs^TA−^ differentiated to UC-like cells for 3 and 7 days. FITC-dextran was incubated with cells grown on the insert, and media in the bottom chamber was analyzed after 3 h. Results were plotted as a percentage relative to the “no cell” control. Positive leakage prevention was observed in both urothelial-induced USCs^TA+^ and USCs^TA−^ when compared to the negative control SMCs. **(B)** Morphology of USCs after induction with SMC induction media for 14 days. Cells were spindle-shaped after induction. Human ureter SMCs served as the positive control. Smooth muscle-specific proteins (calponin and smoothelin) were detected via immunoblotting. Specific signals (bands) were observed in lanes with proteins from the induced USCs^TA+^ and SMC control. **(C)** USCs cultured in osteogenic differentiation media were evaluated for osteogenic cell lineages. Osteogenic differentiation-day 21, positive von Kossa staining for bone minerals was observed in the induced USCs^TA+^ and USCs^TA−^ D). Summary of the differentiation capacity of USC^TA+^ vs USC^TA−^ clones. A35-clone (USCs^TA+,^ RTA: 45.60) and A42 clone (USCs^TA−^, RTA: negative) were both isolated from one donor (male, 27 year-old); J29 (USCs^TA+^, RTA:46.6) and J18 (USCs^TA−^, RTA: negative) were isolated from another donor (male, 55 year-old).

The USC^TA+^ and USCs^TA−^ paired clones from a single donor were tested in parallel to prevent other factors from affecting the results of the comparison. To test the difference in differentiation capability between USCs^TA+^ and USCs^TA−^, both USCs^TA+^ and USCs^TA−^ (p3) were induced to osteogenic, myogenic, and uroepithelial differentiation (Table 4). Both the USCs^TA+^ clone A35 that was isolated from a 27 year-old male donor, and the USCs^TA−^ clone A42 that was isolated from the same donor (p3) differentiated into the smooth muscle lineage and the urothelial lineage (Figures 7A,B). Urothelial-differentiated cells developed a cobblestone-like morphology (Figure 7Ai) and expressed the urothelial-specific proteins uroplakin-Ia and uroplakin-III and the generic epithelial cell markers CK7, CK13, and AE1/AE3 (Figure 7Aii). Furthermore, the expression of these proteins was significantly higher in the UC-induced USCs^TA+^ clones [A35 and J29 (from another male aged 55 years)] than in the UC-induced USCs^TA−^ clones (A42 and J18 [from the same male aged 55 years as clone J29]) and uninduced USC clones. In an assay of cellular barrier function, the UC-induced USCs^TA+^ clone-A35 showed increased expression of specific tight junction protein markers (E-cadherin) compared to the UC-induced USCs^TA−^clone-A42 and uninduced USC clones (Figure 7Ai). In barrier function assays, both the urothelial-differentiated USCs (USC^TA+^ 41.0% ± 1.7% and USC^TA−^ 44.8% ± 0.4%) showed significant reduction in leakage of fluorescent tracer through the insert *in vitro* (*p <* 0.01 and *p <* 0.001, respectively), which means lower permeability and higher tight junction property, compared to the non-differentiated USCs (USC^TA+^ 63.1% ± 4.3%, USC^TA−^ 62.2% ± 1.1%) (Figure 7Aiii) at day 3; and similar results received at day 7 (*p <* 0.01), the leakage percentage of urothelial-differentiated USCs (USC^TA+^-A35 35.5% ± 1.2% and USC^TA−^-A42 38.9% ± 4.3%), less than the non-differentiated USCs (USC^TA+^-A35 54.8% ± 2.6%, USC^TA−^-A42 59.6% ± 0.5%). However, there was only a slight difference in leakage protection at day 3 between the UC-induced USC^TA+^-A35 and USC^TA−^-A42 clones (*p <* 0.05). In addition, no significant differences between them, although reduce in leakage, were noted in UC-induced USC^TA+^-A35 clone on day 7.

**TABLE 4:** Induced and non-induced multipotent differentiation potential of USCs.

	Positive control	Induced USC^TA+^	Non-induced USC^TA+^	Induced USC^TA−^	Non-induced USC^TA−^
Myogenic differentiation	(4 +) SMC	(3 +) in 1/2	0/1	0/2	0/1
Urothelial differentiation	(4 +) UC	(3 + ∼ 4 +) in 2/4	0/1	0/2	0/1
Osteogenic differentiation	(4 +) ASC	(2 + ∼ 3 +) in 2/2	N/A	0/2	N/A

Abbreviation; SMC, smooth muscle cells; UC, urothelial cells; ASC, adipose-derived stem cells. Notes: 1+, 1–25% differentiated cells; 2+, 26–50% differentiated cells. 3+, 51–75% differentiated cells. 4+, >75% differentiated cells.

Both myogenic differentiated USCs^TA+^ and USCs^TA−^ became elongated and spindle-shaped (Figure 7Bi) and expressed smoothelin, a smooth muscle-specific protein marker, and calponin (Figure 7Bii). Moreover, the expression of smoothelin and calponin of SMC- differentiated USCs^TA+^-A35 were higher than the SMC-differentiated USCs^TA−^-A42 confirmed by western blotting (Figure 7Bii). Finally, both USCs^TA+^-A35 and USCs^TA−^-A42 were also induced to differentiate into the osteogenic lineage using our previous protocols. USCs^TA+^-A35 could be induced to osteocytes but not USCs^TA−^-A42, as confirmed by Alizarin S Red staining (Figure 7C). Moreover, both USCs^TA+^-A35 and USCs^TA−^-A42 were difficult to differentiate into adipocytes, as evidenced by Oil Red-O staining (data not shown).

### 3.5 Karyotype Remains Stable in USCs^TA+^

To investigate the potential susceptibility of USCs^TA+^ to malignant transformation, cells were tested via cytogenetic analysis, agar culture *in vitro*, and teratoma formation *in vivo*, and the results were compared to those of USCs^TA−^ and controls.

**FIGURE 8:**
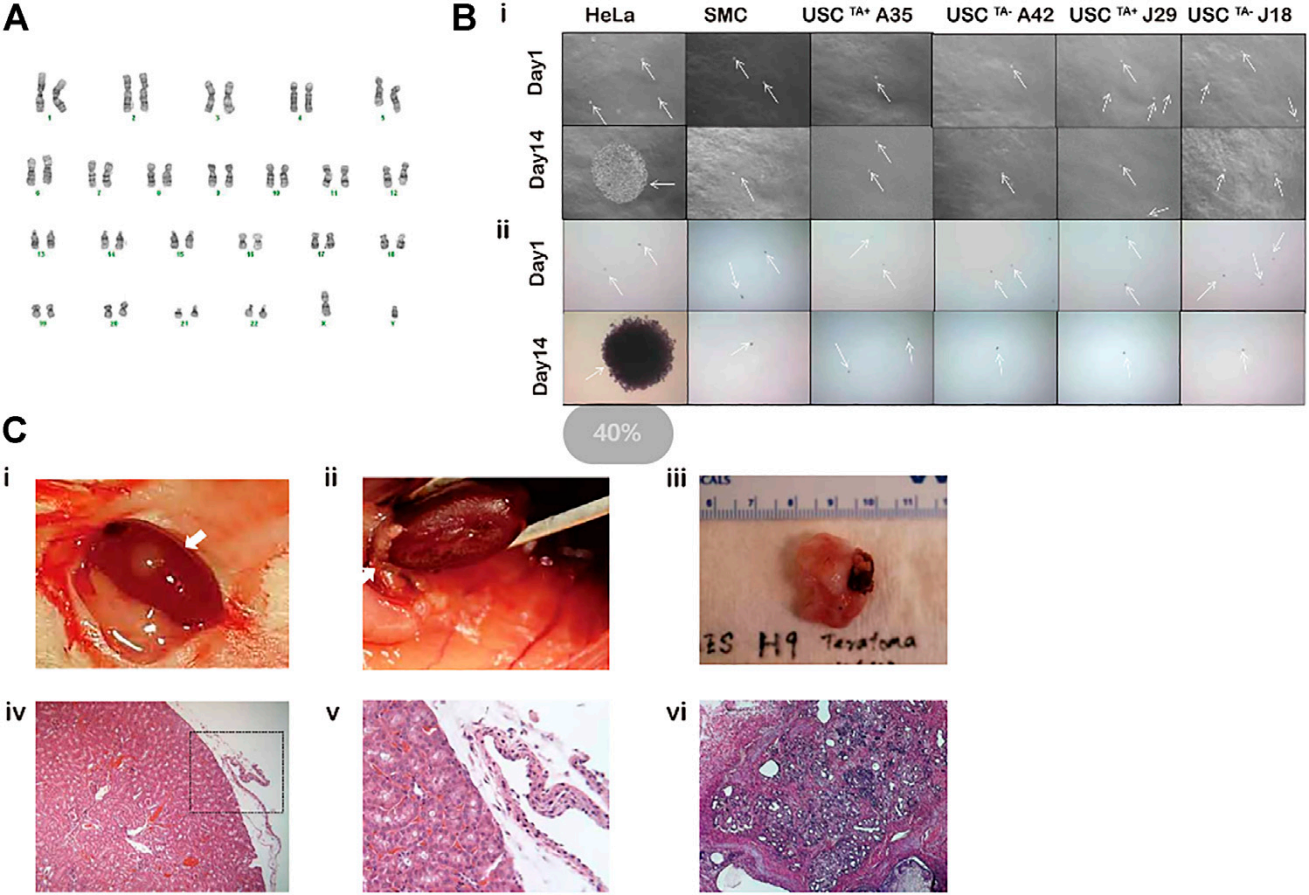
Spontaneous transformation assays of USC^TA+^ clones. **(A)** Karyotypes of USC^TA+^ vs. USC^TA−^ clones. Both USC^TA+^ (*n* = 2, A35, RTA:45.6; and J29, RTA:46.6) and USC^TA−^ (*n* = 2, A42 and J18) clones in the early passage (p4) and late passage (TA + clone in p12, TA-clone at p8) displayed normal complement of diploid (2n = 46). **(B)** *In vitro* agar assay of USCs. The size of the cell clones (seeded at a density of 5,000 cells/well) of USC^TA+^ clones (A35 and J29) and USC^TA−^ clones (A42 and J18) remained the same between days 1 and 14 after plating on soft agar gel. However, clone size of the cancer cell line HeLa cells, as the positive control, at 14 days were significantly increased compared to that at the first day. Normal bladder smooth muscle cell clones were used as the negative control. Images were captured using a phase contrast microscope. Cell clones were stained with nitro blue tetrazolium and photographed using bright field soft agar assays for anchorage-independent cell growth of USC^TA+^ *in vitro*. **(C)** *In vitro* transformation assay of USCs. No teratoma formation was observed under the microscope 2 months after USC^TA+^ (two million cells/graft, *n* = 2, white arrow) were implanted into the capsules of the kidneys of NSG mice. There was no teratoma observable grossly (i, ii) or microscopically (iii, iv). In contrast, H9 cells as the positive control formed derivatives of all the three embryonic germ layers (v, vi). A35-clone (USCs^TA+^, RTA: 45.60) and A42 clone (USCs^TA−^, RTA: negative) were both isolated from one donor (male, 27 year-old); J29 (USCs^TA+^, RTA:46.6) and J18 (USCs^TA−^, RTA: negative) were isolated from another donor (male, 55 year-old).

Both TA+ and TA- USC clones in the early (p4) and late passage (USCs^TA+^-A35, J29 clone in p12 and USCs^TA−^-A42, J18 clone at p8) displayed a normal karyotype of 1 X and 1 Y chromosome, as expected for a male donor, and a normal diploid (2n = 44) complement of autosomes and a pair of sex chromosomes (Figure 8A; Table 5). No multiploidy or obvious chromosomal rearrangements in metaphase were detected by Giemsa bandings at p4 or late passage of both USC clones.

**TABLE 5:** Conventional karyotypes of USC^TA+^ and USC^TA-^ clones in the 20- and 50-years age groups at early and late passages.

	Age at 20s group		Age at 50s group	
	USC clones	Karyotypes	USC clones	Karyotypes
**USC** ^**TA+**^	A35 (p4)	46, XY	J29 (p4)	46, XY
	A35 (p12)	46, XY	J29 (p12)	46, XY
**USC** ^**TA−**^	A42 (p4)	46, XY	J18 (p4)	46, XY
	A42 (p8)	46, XY	J18 (p8)	46, XY

Notes**:** A35-clone (USCs^TA+,^ RTA: 45.60) and A42 clone (USCs^TA**−**^, RTA: negative) were both isolated from one donor (male, 27-year-old); J29 (USCs^TA+^, RTA:46.6) and J18 (USCs^TA**−**^, RTA: negative) were isolated from another donor (male, 55 year-old).

### 3.6 No Tumorigenic Transformation of USCs^TA+^ Was Observed

USC^TA+^ clones-A35, J29 remained the same size on day 14 as that on day 1, after being cultured in soft agar, like colonies of SMCs. However, HeLa cancer cells formed large colonies on day 14 (Figure 8Bi). These single cells (SMC and USC) or colonies (HeLa cancer cells) were viable, which was confirmed by NBT staining (Figure 8Bii). Furthermore, no teratoma was formed when all USCs^TA+^-A35 were implanted in the subcapsular region of the kidney of NSG mice after 8 weeks. All human ES cell lines (H9) formed derivatives of the three embryonic germ layers (Figure 8C).

## 4 Discussion

We characterized the stemness features of human USCs, including long-term survival with self-renewal capacity, multi-lineage differentiation, MSCs surface markers, expression of telomere maintenance mechanisms (TA) in *in vitro* culture time frames, and capacity to form teratomas. USC clones in the same individual urine sample displayed telomere heterogeneity, which could be due to USCs at different stages of the telomerase activation processes.

Stemness refers to the molecular processes underlying the fundamental stem cell properties of self-renewal and the generation of differentiated daughter cells. The stemness properties of adult-derived stem cells decline after birth, compared to those of ESCs (Hofmeister et al., 2015). Most human somatic or stem cells do not express OCT4/SOX2/KLF4/MYC. Forced expression of OCT4/SOX2/KLF4/MYC in somatic cells such as fibroblasts can reprogram cells to a pluripotent stem cell fate. USCs are multipotent, rather than pluripotent, and express low levels of OCT4/SOX2/KLF4/MYC (Bharadwaj et al., 2013). Our previous studies demonstrated that USCs possess limited stemness properties including self-renewal and multiple differentiation capacity but do not induce teratoma formation. This is different from iPSC that have higher expression levels of OCT4/SOX2/KLF4/MYC and form teratoma. In summary, USCs are multipotent and thus do not express the higher levels of OCT4/SOX2/KLF4/MYC that are observed in pluripotent stem cells.

Two methods are predominantly used to track the *in vitro* age of a cell culture or cell proliferation capacity. 1) The passage number implies the number of times a cell has been passaged, which is most commonly used in the laboratory. However, the cell passage number is imprecise because different laboratories may use different initial cell seeding densities, which affects the number of times cells divide in culture. 2) The PD indicate the number of cell generations the cell lineage has undergone—the number of times the cell population has doubled. PD of primary cells is a better practice for reporting cellular age *in vitro*, which is often used to set an acceptable upper limit for cell production, or the maximum number of cells generated in culture. In this study, we used both terms (passage number and PD number) to present cell lifespan, and there was good agreement between the two measures. USCs^TA+^ with higher TA could reach higher PD number or passage number, a shorter DT which indicates faster cell division or more rapid cell growth, and better proliferation capacity which indicates more cells generated than those of USCs^TA+^ with lower TA strength and USCs^TA−^. Furthermore, the TA of USCs^TA+^ declined with passage; thus, the proliferation potential with PD and DT of USCs gradually decreased, with the cells finally reaching senescence within 8 weeks in 2D culture and 10 weeks in 3D culture models (data not shown).

Telomerase is activated and maintains cellular immortality in ESCs or iPSCs, which plays an import role such as to protect the genome from nucleolytic degradation, unnecessary recombination, repair, and intrachromosomal fusion (Hiyama and Hiyama, 2007); however, the level of TA is low or absent in most MSCs and ASCs (Zimmermann et al., 2003; Hiyama and Hiyama, 2007) regardless of their proliferative capacity. Numbers of BMSCs are low in bone marrow nucleated cells with a frequency of colony-forming unit-fibroblasts (CFU-F) of 1: 35,700 (Lu et al., 2006). In addition, small amounts of stem cells are mixed with a large amount of stromal cells in the bone marrow, which makes it challenging to isolate true stem cells and measure their levels of TA. In contrast, USCs start with a single stem cell that forms cell clones and expands to a large number of stem cells of which most display TA. There are a couple of studies comparing hUSC to hBMSC (Sun et al., 2021) and ASC (Kang et al., 2015). In *in vitro* experiments, hUSC presented with better capacity for proliferation than hBMSC, while hBMSC had greater chondrogenic ability than hUSC. However, hUSC and hBMSC had similar cartilage repair effects *in vivo*. Results indicated that hUSC can be a stem cell alternative for cartilage regeneration, provide a powerful platform for cartilage tissue engineering, and clinical transformation (Sun et al., 2021). Similar outcomes are achieved in studies of comparison between hUSC and hASC (Kang et al., 2015). TA levels can be detected or consistently expressed in most human USCs (>70%) obtained from healthy middle-aged donors, although levels of this enzyme were lower than those of ESC. Most USCs express TA however, along with the aging process in individuals, a reduction in USC regenerative capacity occurs in the 50s age group, which also means a decrease in cell proliferation capacity along with the reduced number of USCs^TA+^ (59%), and relatively lower PD and DT and a decline in telomere reserve with associated lower TA. Furthermore, the number of USCs^TA+^ declined with increasing passage during cell proliferation. Thus, the levels of TA decreased with an increase in donor age and cell passage but did not show an increase again after long-term culture, demonstrating the safety of USC implantation with retaining chromosome stable and no oncological transformation. The TA of USCs^TA+^ declined with passage *in vitro* or throughout individual age, providing a mechanism that restricts cell over-proliferation and any tumor development.

Human MSCs as a heterogeneous cell population are confirmed by a set of cell surface makers, instead of a single marker. MSCs often maintain their immunophenotypic characteristics stably throughout the culture term. The ASCs did not show immunophenotypic alterations with passage and retained a consistently high expression level of MSC surface markers and were negative for HSC at early (p4) and late passage (p8) markers (Nava et al., 2015a). In this study, we observed there were USCs at different stages of activation. One was the activating USCs with positive telomerase activity; one is activated cells (relatively old) with negative TA; but both USCs^TA+^ and USCs^TA−^ in p4 showed the most MSC surface markers (CD44, CD73, CD90, CD105, CD146, and HLA-A, B, C) and lacked hematopoietic markers (CD25. CD31, CD34, CD45, HLA-DR/DP/DQ, and HLA-G). Our data demonstrated that USCs are a good starting population because of their lack of immunological reactivity. Importantly, through the most exhaustive head-to-head characterization of multiple clones that each start a single USC clonal population from multiple donors, these results allow us to highlight the intrinsic differences between commonly used starting cell sources.

In addition, CD105 is one unique marker associated with differentiation potential (Dominici et al., 2006). For example, CD105^+^ ASCs were more prone to differentiation into chondrocytes than CD105^−^ ASCs (Ishimura et al., 2008; Jiang et al., 2010). CD105^+^ MSCs were also more efficient in the infarcted heart (Gaebel et al., 2011), with stronger proliferative and colony formation abilities than CD105^−^ MSCs (Lv et al., 2012). However, CD105^−^ ASCs were more osteogenic (Jiang et al., 2010) and showed strong immunomodulation capacity (Anderson et al., 2013). Interestingly, five of the six USC^TA+^ clones strongly expressed CD105, and four of the six USC^TA−^ clones either did not express CD105 or were weakly positive. Compared to other CD markers, CD105^+^ appeared to correlate with stemness. The correlation between CD105^+^ USC and USC^TA+^ of stem cells appears to have different capabilities. It is worth further investigation into the mechanism of correlation in both TA and CD105 marker cells with such different capabilities.

Telomerase and telomeres are strongly associated with cell renewal and proliferation, but it is controversial whether telomerase is associated with cell differentiation in MSCs (Zimmermann et al., 2003; Hiyama and Hiyama, 2007). The epigenetic nature of telomeres appears to depend on different human cell linages (Dogan and Forsyth, 2021). In adult human stem cells, both BMSCs and ASCs lack telomerase, but can retain their functional characteristics and multiple differentiation potential (Zimmermann et al., 2003; Hiyama and Hiyama, 2007). Interestingly, overexpression of telomerase resulted in telomere elongation, and TERT-transfected cells continued to proliferate and formed bone *in vivo* (Simonsen et al., 2002). However, mouse MSCs with their TA knocked down failed to differentiate into adipocytes or chondrocytes, even at early passage (Liu et al., 2004). Similarly, increased TA enhanced self-renewal ability, proliferation, and differentiation efficiency in TERT-overexpressing ES cells (Armstrong et al., 2005). High TA or the expression of TERT can therefore be regarded as a marker of undifferentiated ES cells. Downregulation of TA in differentiating EC cells was reported to be closely correlated with histone deacetylation and DNA methylation of the TERT gene (Lopatina et al., 2003). In this study, USCs^TA+^ performed better in terms of multiple differentiation capacity in osteogenic, myogenic, and uroepithelial differentiation than USCs^TA-^, indicating that telomerase is required for not only cell proliferation but also multiple differentiation in human USCs, which is a guarantee for future studies to determine whether USC^TA+^ induce better *in vivo* tissue regeneration than USC^TA−^.

As telomerase is related to both normal stem cells and tumor stem cells, it is necessary to determine the alteration of karyotypes, with the *in vitro* agar assay and the *in vivo* risk of tumor formation of telomerase-positive cells for their safe transplantation application. Giemsa-based chromosomal banding and staining techniques are important for cytogenetics. USCs maintain a normal diploid chromosome recognized as 46 during long-term culture or overexpansion for up to p16 or 68 PD (2^68^). Cytogenetic analyses showed USCs can safely expand *in vitro* (p4, p8, and p16) with no sign of immortalization or development of chromosomal abnormalities.

Cloning techniques with semisolid medium, such as agar gel for evaluating cell growth, are commonly used to study the biology of cancer cells due to their anchorage-independent growth requirements. The ability of cancer cells to proliferate without firm attachment (i.e., anchorage independence) is one of the best *in vitro* indicators of tumorigenicity. Importantly, USCs^TA+^ do not present a propensity for spontaneous oncogenic transformation 60 days after *in vivo* implantation. The tumorigenic potential of USCs^TA+^ was not found *in vitro* or *in vivo*. Thus, USCs as a new source of seed cells, which are non-invasive, highly proliferative, and abundant, can be used for tissue engineering and regenerative medicine.

TA appears to be related to the stemness of USCs (Table 6). USCs^TA+^ survived for a significantly longer period with intact morphological appearance, rapid proliferation, and ample cell generation, and possessed more potent differentiation capacity than USCs^TA−^. Both human USCs^TA+^ and USCs^TA−^ can be safely expanded *in vitro* maintaining normal karyotype and showed tumor-free formation after *in vivo* implantation, which makes them appropriate sources for cell-based therapies. Thus, TA could be an independent predictive factor for the regenerative capacity of USCs. In addition, human USCs^TA+^ provides sufficient cell numbers for drug testing. TA is a good indicator of stemness (cell renewal and differentiation potential) of human adult stem cells, but a collection of primary cultured cells is required, which is cumbersome. Therefore, simpler and low-cost methods of measuring TA and telomeres in stem cells are highly desired.

**TABLE 6:** Feature summary of human USCs with telomerase activity in USC^TA+^ and USC^TA**−**^ clones.

	USC^TA+^	USC^TA−^
Cell renewal		
- Population doubling	Higher	Lower
- Doubling time	Shorter	Longer
- Passages	More	More
Cell differentiation		
- Osteogenic makers	Strong positive	Negative
- Smooth muscle makers	Strong positive	Weak expression
- Urothelial markers	Strong positive	negative
MSC markers		
- CD44, CD73, CD90, CD146,	Strong positive	Strong positive
- CD25, CD31, CD34, CD45, CD117, CD140b HLA-DR/DP/DQ, HLA-G	Negative	Negative
- CD105 Strong positive	4/6	2/6
Age-dependent		
- 20–40s	73–77% of USC	23–27%
- 50s	59% of USC	41%
**Safety**		
- Chromosome stability	Yes	Yes
- Tumor Colone formation in aga	Negative	Negative
- Teratoma formation *in vivo*	Negative	Negative

## 5 Conclusion

This study demonstrated that human primary urinary stem cells with positive TA act as a distinct subpopulation with potential regeneration capacity in both cell proliferation and multiple differentiation. USCs^TA+^ can more efficiently give rise to osteogenic, skeletal myogenic, smooth muscle, and urothelial cell lines than USCs^TA−^. Importantly, despite that USCs display TA, they do not form teratoma, which provides a safe cell source for clinical application. In addition, the number of USCs^TA+^ decline with increasing age. Future investigations should focus on understanding the role that physiological factors play in regulating both the temporal pattern of USCs^TA+^ and their influence on the ability of these cells to participate in better tissue repair. Determining the requirements for the effect of TA on the paracrine effect of USC has important implications for understanding the anti-inflammatory, fibrosis inhibition, and redox effect of USCs^TA+^. It will be beneficial to better understand alterations in this cell subpopulation throughout the human lifespan, and how they translate into, aging, renal dysfunction, drug-induced nephrotoxicity, or cancer, among others.

## Data Availability

The original contributions presented in the study are included in the article/Supplementary Material, further inquiries can be directed to the corresponding author.
